# (*S*)-2-Ammonio-3-(4-nitro­phen­yl)propanoate monohydrate

**DOI:** 10.1107/S1600536808011203

**Published:** 2008-05-03

**Authors:** Wei Dai, Da-Wei Fu

**Affiliations:** a Ordered Matter Science Research Center, College of Chemistry and Chemical Engineering, Southeast University, Nanjing 210096, People’s Republic of China

## Abstract

The title compound, C_9_H_10_N_2_O_4_·H_2_O, exists as a zwitterion with a deprotonated carboxyl group and a protonated amino group. The crystal packing is stabilized by N—H⋯O and O—H⋯O hydrogen bonds, building sheets parallel to the (001) plane. The absolute configuration was deduced from the synthetic pathway.

## Related literature

For details of α-amino acids as precursors for the synthesis of novel biologically active compounds, see: Lucchese *et al.* (2007 ▶); Arki *et al.* (2004 ▶); Hauck *et al.* (2006 ▶); Azim *et al.* (2006 ▶).
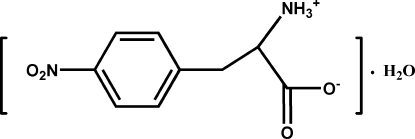


## Experimental

### 

#### Crystal data

C_9_H_10_N_2_O_4_·H_2_O
*M* *_r_* = 228.21
Orthorhombic, 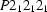

*a* = 5.3141 (8) Å
*b* = 6.2823 (7) Å
*c* = 30.752 (4) Å
*V* = 1026.7 (2) Å^3^
*Z* = 4
Mo *K*α radiation
μ = 0.12 mm^−1^
*T* = 293 (2) K
0.25 × 0.20 × 0.20 mm

#### Data collection

Rigaku Mercury2 diffractometer
Absorption correction: multi-scan (*CrystalClear*; Rigaku, 2005 ▶) *T*_min_ = 0.970, *T*_max_ = 0.974
10728 measured reflections
1466 independent reflections
1261 reflections with *I* > 2σ(*I*)
*R*_int_ = 0.046

#### Refinement

*R*[*F* ^2^ > 2σ(*F* ^2^)] = 0.043
*wR*(*F* ^2^) = 0.113
*S* = 1.09
1466 reflections
146 parameters
H-atom parameters constrained
Δρ_max_ = 0.25 e Å^−3^
Δρ_min_ = −0.22 e Å^−3^

### 

Data collection: *CrystalClear* (Rigaku, 2005 ▶); cell refinement: *CrystalClear*; data reduction: *CrystalClear*; program(s) used to solve structure: *SHELXS97* (Sheldrick, 2008 ▶); program(s) used to refine structure: *SHELXL97* (Sheldrick, 2008 ▶); molecular graphics: *SHELXTL* (Sheldrick, 2008 ▶); software used to prepare material for publication: *SHELXL97*.

## Supplementary Material

Crystal structure: contains datablocks I, global. DOI: 10.1107/S1600536808011203/dn2340sup1.cif

Structure factors: contains datablocks I. DOI: 10.1107/S1600536808011203/dn2340Isup2.hkl

Additional supplementary materials:  crystallographic information; 3D view; checkCIF report

## Figures and Tables

**Table 1:** Hydrogen-bond geometry (Å, °)

*D*—H⋯*A*	*D*—H	H⋯*A*	*D*⋯*A*	*D*—H⋯*A*
N2—H2*B*⋯O1*W*	0.89	1.88	2.760 (3)	168
N2—H2*A*⋯O1*W*^i^	0.89	2.43	3.029 (3)	125
N2—H2*A*⋯O4^ii^	0.89	2.29	2.913 (3)	127
N2—H2*C*⋯O3^iii^	0.89	1.92	2.797 (3)	166
O1*W*—H1*WB*⋯O3^iv^	0.85	2.23	2.764 (3)	121
O1*W*—H1*WC*⋯O4^v^	0.85	2.00	2.740 (3)	146

Symmetry codes: (i) 
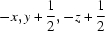
; (ii) 
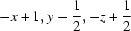
; (iii) 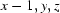; (iv) 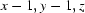; (v) 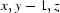.

**Fig. 1.**
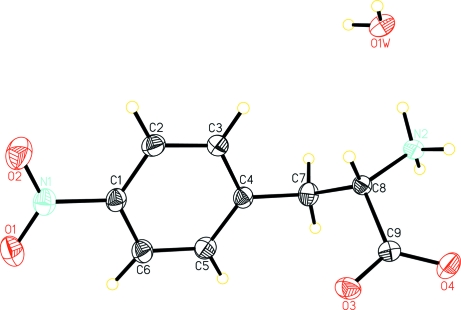
A view of the title compound with the atomic numbering scheme. Displacement ellipsoids were drawn at the 30% probability level.

**Fig. 2.**
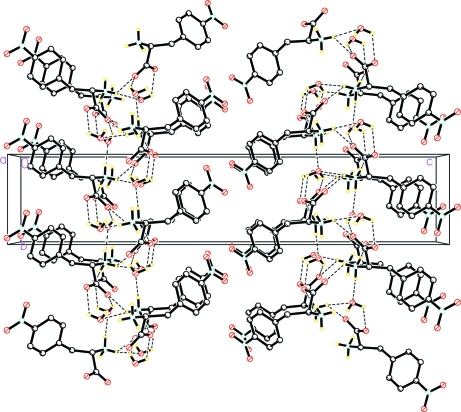
The crystal packing of the title compound viewed along the a axis and all hydrogen atoms not involved in hydrogen bonding (dashed lines) were omitted for clarity.

## supplementary crystallographic information

### Comment

α-Amino acids are important molecules due to their pharmacological properties. Recently, there has been an increased interest in the enantiomeric preparation of α-amino acids as precursors for the synthesis of novel biologically active compounds (Lucchese *et al.*, (2007); Arki *et al.*, (2004); Hauck *et al.*, (2006); Azim *et al.*, (2006)).Here we report the synthesis and crystal structure of the title compound.

The title compound exists as a zwitter ion with a deprotonated carboxyl group and a protonated amino group (Fig. 1). It crystallizes with one water molecule in the asymmetric unit. The crystal packing is stabilized by N—H···O and O—H···O hydrogen bonds building sheets parallel to the (0 0 1) plane (Table 1, Figs. 2).

The *S* absolute configuration at C8 is deduced from the synthetic pathway.

### Experimental

Under nitrogen protection, 2-amino-3-phenylpropanoic acid (30 mmol), nitric acid (50 mmol) and sulfuric acid (20 mmol) were added in a flask. The mixture was stirred at 110 °C for 3 h. The resulting solution was poured into ice water (100 mL), then filtered and washed with distilled water. The crude product was recrystallized with distilled water to yield colorless block-like crystals, suitable for X-ray analysis.

### Refinement

All H atoms attached to C atoms and N atom were fixed geometrically and treated as riding with C—H = 0.98 Å (methine), 0.97 Å (methylene), 0.93 Å (aromatic) and N—H = 0.89 Å with U_iso_(H) = 1.2U_eq_(C) or U_iso_(H) = 1.5U_eq_(N). H atoms of water molecule were located in difference Fourier maps and included in the subsequent refinement using restraints (O-H= 0.85 (1)Å and H···H= 1.39 (2)Å) with U_iso_(H) = 1.5U_eq_(O). In the last stage of refinement they were treated as riding on their parent O atom.

In the absence of significant anomalous scattering, the absolute configuration could not be reliably determined by the X-ray analyses and then the Friedel pairs were merged and any references to the Flack parameter were removed.

### Crystal data

**Table:**

C_9_H_10_N_2_O_4_·H_2_O	*F*_000_ = 480
*M**_r_* = 228.21	*D*_x_ = 1.476 Mg m^−^^3^
Orthorhombic, *P*2_1_2_1_2_1_	Mo *K*α radiation λ = 0.71073 Å
Hall symbol: P 2ac 2ab	Cell parameters from 1470 reflections
*a* = 5.3141 (8) Å	θ = 3.0–27.5º
*b* = 6.2823 (7) Å	µ = 0.12 mm^−^^1^
*c* = 30.752 (4) Å	*T* = 293 (2) K
*V* = 1026.7 (2) Å^3^	Block, colourless
*Z* = 4	0.25 × 0.20 × 0.20 mm

### Data collection

**Table:**

Rigaku Mercury2 diffractometer	1466 independent reflections
Radiation source: fine-focus sealed tube	1261 reflections with *I* > 2σ(*I*)
Monochromator: graphite	*R*_int_ = 0.046
Detector resolution: 13.6612 pixels mm^-1^	θ_max_ = 27.8º
*T* = 293(2) K	θ_min_ = 3.3º
ω scans	*h* = −6→6
Absorption correction: multi-scan(CrystalClear; Rigaku, 2005)	*k* = −8→8
*T*_min_ = 0.970, *T*_max_ = 0.974	*l* = −40→40
10728 measured reflections	

### Refinement

**Table:**

Refinement on *F*^2^	Secondary atom site location: difference Fourier map
Least-squares matrix: full	Hydrogen site location: inferred from neighbouring sites
*R*[*F*^2^ > 2σ(*F*^2^)] = 0.043	H-atom parameters constrained
*wR*(*F*^2^) = 0.113	*w* = 1/[σ^2^(*F*_o_^2^) + (0.0645*P*)^2^ + 0.0774*P*] where *P* = (*F*_o_^2^ + 2*F*_c_^2^)/3
*S* = 1.09	(Δ/σ)_max_ < 0.001
1466 reflections	Δρ_max_ = 0.25 e Å^−^^3^
146 parameters	Δρ_min_ = −0.22 e Å^−^^3^
Primary atom site location: structure-invariant direct methods	Extinction correction: none

### Special details

**Table:**

Geometry. All esds (except the esd in the dihedral angle between two l.s. planes) are estimated using the full covariance matrix. The cell esds are taken into account individually in the estimation of esds in distances, angles and torsion angles; correlations between esds in cell parameters are only used when they are defined by crystal symmetry. An approximate (isotropic) treatment of cell esds is used for estimating esds involving l.s. planes.
Refinement. Refinement of *F*^2^ against ALL reflections. The weighted *R*-factor *wR* and goodness of fit *S* are based on *F*^2^, conventional *R*-factors *R* are based on *F*, with *F* set to zero for negative *F*^2^. The threshold expression of *F*^2^ > σ(*F*^2^) is used only for calculating *R*-factors(gt) *etc*. and is not relevant to the choice of reflections for refinement. *R*-factors based on *F*^2^ are statistically about twice as large as those based on *F*, and *R*- factors based on ALL data will be even larger.

### Fractional atomic coordinates and isotropic or equivalent isotropic displacement parameters (Å^2^)

**Table:**

	*x*	*y*	*z*	*U*_iso_*/*U*_eq_	
O3	0.8183 (3)	0.4808 (3)	0.17549 (6)	0.0391 (4)	
N2	0.2254 (4)	0.2416 (3)	0.20604 (6)	0.0346 (5)	
H2A	0.2535	0.2749	0.2337	0.052*	
H2B	0.1637	0.1101	0.2044	0.052*	
H2C	0.1152	0.3328	0.1947	0.052*	
O4	0.5036 (4)	0.5887 (3)	0.21776 (6)	0.0452 (5)	
O1	1.3141 (4)	−0.0801 (4)	0.00871 (7)	0.0588 (6)	
C1	1.0030 (5)	−0.0711 (4)	0.06148 (7)	0.0342 (6)	
N1	1.2123 (5)	−0.1761 (4)	0.03883 (7)	0.0410 (5)	
C7	0.4109 (5)	0.2328 (4)	0.13231 (8)	0.0369 (6)	
H7A	0.2588	0.1496	0.1284	0.044*	
H7B	0.3801	0.3735	0.1205	0.044*	
C9	0.6059 (5)	0.4594 (4)	0.19280 (7)	0.0301 (5)	
C8	0.4651 (4)	0.2532 (4)	0.18137 (7)	0.0283 (5)	
H8	0.5704	0.1325	0.1901	0.034*	
C3	0.6976 (6)	−0.0771 (4)	0.11762 (8)	0.0391 (6)	
H3	0.6180	−0.1481	0.1403	0.047*	
C5	0.7376 (5)	0.2308 (4)	0.07239 (8)	0.0376 (6)	
H5	0.6850	0.3663	0.0643	0.045*	
C4	0.6219 (5)	0.1297 (4)	0.10701 (7)	0.0327 (5)	
C2	0.8872 (5)	−0.1774 (4)	0.09522 (8)	0.0405 (6)	
H2	0.9366	−0.3146	0.1027	0.049*	
C6	0.9322 (5)	0.1322 (4)	0.04943 (8)	0.0397 (6)	
H6	1.0122	0.2016	0.0266	0.048*	
O2	1.2768 (5)	−0.3521 (4)	0.05090 (7)	0.0589 (6)	
O1W	0.0704 (4)	−0.1769 (3)	0.21144 (6)	0.0468 (5)	
H1WB	0.0482	−0.2262	0.1860	0.070*	
H1WC	0.1824	−0.2497	0.2244	0.070*	

### Atomic displacement parameters (Å^2^)

**Table:**

	*U*^11^	*U*^22^	*U*^33^	*U*^12^	*U*^13^	*U*^23^
O3	0.0300 (9)	0.0402 (10)	0.0470 (10)	−0.0064 (8)	0.0032 (7)	0.0024 (8)
N2	0.0329 (10)	0.0331 (10)	0.0377 (11)	−0.0040 (10)	0.0050 (8)	−0.0008 (9)
O4	0.0382 (10)	0.0407 (10)	0.0568 (11)	−0.0015 (9)	−0.0003 (9)	−0.0179 (9)
O1	0.0581 (13)	0.0682 (14)	0.0501 (12)	0.0028 (12)	0.0185 (10)	−0.0021 (11)
C1	0.0371 (14)	0.0365 (12)	0.0291 (11)	−0.0012 (11)	−0.0015 (10)	−0.0080 (10)
N1	0.0388 (12)	0.0467 (13)	0.0374 (11)	0.0022 (11)	−0.0027 (9)	−0.0075 (10)
C7	0.0366 (13)	0.0428 (13)	0.0311 (12)	0.0021 (12)	−0.0043 (10)	−0.0044 (11)
C9	0.0295 (12)	0.0295 (11)	0.0312 (11)	0.0004 (10)	−0.0057 (9)	0.0031 (10)
C8	0.0274 (11)	0.0284 (11)	0.0293 (11)	−0.0003 (10)	0.0012 (9)	0.0001 (9)
C3	0.0486 (15)	0.0338 (12)	0.0349 (12)	−0.0017 (12)	0.0074 (12)	0.0008 (11)
C5	0.0471 (15)	0.0344 (12)	0.0313 (12)	0.0031 (12)	−0.0031 (11)	0.0021 (10)
C4	0.0348 (13)	0.0351 (12)	0.0283 (11)	−0.0023 (11)	−0.0019 (9)	−0.0050 (10)
C2	0.0537 (16)	0.0315 (12)	0.0364 (12)	0.0009 (12)	0.0017 (12)	0.0013 (11)
C6	0.0473 (15)	0.0415 (14)	0.0304 (11)	0.0002 (12)	0.0038 (12)	0.0001 (11)
O2	0.0594 (14)	0.0560 (12)	0.0612 (13)	0.0206 (12)	0.0009 (12)	−0.0009 (11)
O1W	0.0493 (11)	0.0347 (9)	0.0563 (11)	−0.0032 (9)	−0.0051 (9)	−0.0047 (9)

### Geometric parameters (Å, °)

**Table:**

O3—C9	1.255 (3)	C7—H7B	0.9700
N2—C8	1.484 (3)	C9—C8	1.537 (3)
N2—H2A	0.8900	C8—H8	0.9800
N2—H2B	0.8900	C3—C2	1.374 (4)
N2—H2C	0.8900	C3—C4	1.398 (4)
O4—C9	1.243 (3)	C3—H3	0.9300
O1—N1	1.231 (3)	C5—C4	1.384 (3)
C1—C2	1.379 (4)	C5—C6	1.397 (4)
C1—C6	1.382 (4)	C5—H5	0.9300
C1—N1	1.469 (3)	C2—H2	0.9300
N1—O2	1.216 (3)	C6—H6	0.9300
C7—C4	1.511 (3)	O1W—H1WB	0.8502
C7—C8	1.541 (3)	O1W—H1WC	0.8496
C7—H7A	0.9700		
			
C8—N2—H2A	109.5	N2—C8—C7	109.62 (18)
C8—N2—H2B	109.5	C9—C8—C7	112.6 (2)
H2A—N2—H2B	109.5	N2—C8—H8	108.1
C8—N2—H2C	109.5	C9—C8—H8	108.1
H2A—N2—H2C	109.5	C7—C8—H8	108.1
H2B—N2—H2C	109.5	C2—C3—C4	121.3 (2)
C2—C1—C6	121.9 (2)	C2—C3—H3	119.3
C2—C1—N1	118.5 (2)	C4—C3—H3	119.3
C6—C1—N1	119.6 (2)	C4—C5—C6	120.9 (2)
O2—N1—O1	123.5 (3)	C4—C5—H5	119.5
O2—N1—C1	118.5 (2)	C6—C5—H5	119.5
O1—N1—C1	118.0 (2)	C5—C4—C3	118.6 (2)
C4—C7—C8	113.6 (2)	C5—C4—C7	121.9 (2)
C4—C7—H7A	108.8	C3—C4—C7	119.5 (2)
C8—C7—H7A	108.8	C3—C2—C1	118.8 (2)
C4—C7—H7B	108.8	C3—C2—H2	120.6
C8—C7—H7B	108.8	C1—C2—H2	120.6
H7A—C7—H7B	107.7	C1—C6—C5	118.4 (2)
O4—C9—O3	125.8 (2)	C1—C6—H6	120.8
O4—C9—C8	118.6 (2)	C5—C6—H6	120.8
O3—C9—C8	115.5 (2)	H1WB—O1W—H1WC	109.4
N2—C8—C9	110.03 (19)		

### Hydrogen-bond geometry (Å, °)

**Table:**

*D*—H···*A*	*D*—H	H···*A*	*D*···*A*	*D*—H···*A*
N2—H2B···O1W	0.89	1.88	2.760 (3)	168
N2—H2A···O1W^i^	0.89	2.43	3.029 (3)	125
N2—H2A···O4^ii^	0.89	2.29	2.913 (3)	127
N2—H2C···O3^iii^	0.89	1.92	2.797 (3)	166
O1W—H1WB···O3^iv^	0.85	2.23	2.764 (3)	121
O1W—H1WC···O4^v^	0.85	2.00	2.740 (3)	146

Symmetry codes: (i) −*x*, *y*+1/2, −*z*+1/2; (ii) −*x*+1, *y*−1/2, −*z*+1/2; (iii) *x*−1, *y*, *z*; (iv) *x*−1, *y*−1, *z*; (v) *x*, *y*−1, *z*.
